# γ2 GABA_A_R Trafficking and the Consequences of Human Genetic Variation

**DOI:** 10.3389/fncel.2018.00265

**Published:** 2018-08-23

**Authors:** Joshua M. Lorenz-Guertin, Matthew J. Bambino, Tija C. Jacob

**Affiliations:** Department of Pharmacology and Chemical Biology, School of Medicine, University of Pittsburgh, Pittsburgh, PA, United States

**Keywords:** GABA_A_ receptor, trafficking, genetic variation, human, epilepsy, imaging

## Abstract

GABA type A receptors (GABA_A_Rs) mediate the majority of fast inhibitory neurotransmission in the central nervous system (CNS). Most prevalent as heteropentamers composed of two α, two β, and a γ2 subunit, these ligand-gated ionotropic chloride channels are capable of extensive genetic diversity (α1-6, β1-3, γ1-3, δ, 𝜀, 𝜃, π, ρ1-3). Part of this selective GABA_A_R assembly arises from the critical role for γ2 in maintaining synaptic receptor localization and function. Accordingly, mutations in this subunit account for over half of the known epilepsy-associated genetic anomalies identified in GABA_A_Rs. Fundamental structure–function studies and cellular pathology investigations have revealed dynamic GABA_A_R trafficking and synaptic scaffolding as critical regulators of GABAergic inhibition. Here, we introduce *in vitro* and *in vivo* findings regarding the specific role of the γ2 subunit in receptor trafficking. We then examine γ2 subunit human genetic variation and assess disease related phenotypes and the potential role of altered GABA_A_R trafficking. Finally, we discuss new-age imaging techniques and their potential to provide novel insight into critical regulatory mechanisms of GABA_A_R function.

## Introduction

**FIGURE 1:**
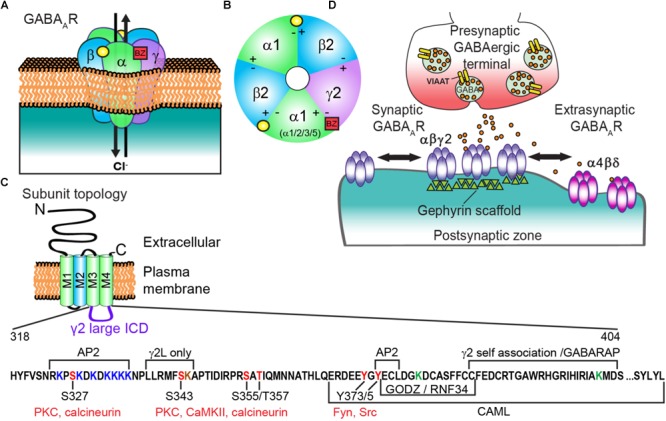
Generic GABA_A_R structure and subunit topology; and regulatory sites of the γ2 intracellular domain (ICD). **(A)** GABA_A_R heteropentamer composed of αβγ subunits. Binding of the neurotransmitter GABA (yellow circle) at the αβ interface triggers ion channel opening and allows the rapid influx of Cl^-^ and membrane hyperpolarization in the mature nervous system. **(B)** Extracellular representation of the most prevalent cortical receptor subtype composed of α1β2γ2 subunits showing all five subunits contributing to the central ion pore and the general binding sites of GABA (yellow circle) and benzodiazepines (BZs) (red square). BZs bind at the interface of an α1/2/3/5 and γ subunit. **(C)** All subunits have a common topology including an extracellular N-terminal domain (ECD), short C-terminal tail, and four transmembrane regions (M1-4) which compose the transmembrane domain (TM). M2 (blue) contributes to formation of the receptor ion channel pore, while the ICD between M3 and M4 contains sites of phosphorylation and protein interactions that modulate channel function and/or trafficking. The γ2 L isoform intracellular domain (ICD = AA 318-404, residue numbering does not include signal peptide) is shown here with identified regulatory sites and regions of protein interaction. Seven lysine residues (red) contribute to γ2-containing GABA_A_R ubiquitination and endo-lysosomal targeting in HEK cells, with mutation of three additional lysine residues needed to block receptor downregulation by E3 ligase RNF34 overexpression (ICD green Ks and K259 in smaller M1-M2 loop not shown in diagram). Note the γ2L specific K344 residue (brown) has not been tested in ubiquitination studies **(D)** GABA_A_Rs composed of α(1-3)βγ subunits are largely synaptically localized via gephyrin interactions and contribute to phasic currents, whereas α(4 or 6)βδ receptors are extrasynaptic and generate tonic current.

The adult central nervous system (CNS) is critically dependent on fast inhibitory neurotransmission evoked by GABA_A_ receptors (GABA_A_Rs). GABA_A_Rs are ligand-gated ionotropic chloride (Cl^-^) channels ubiquitously expressed throughout the CNS that play a fundamental role in restraining and sculpting neuronal activity. Disruptions in GABA_A_R dependent neurotransmission leads to insufficient inhibitory effects throughout the brain, contributing to the pathogenesis of epilepsy, neurodevelopmental disorders, depression, schizophrenia and stroke (Hines et al., 2012). Activation of GABA_A_Rs by the neurotransmitter GABA induces ion channel opening, Cl^-^ influx, and subsequent membrane hyperpolarization. These heteropentameric structures are predominantly composed of two α (α1-6), two β (β1-3), and either a γ (γ1-3) or a δ subunit (Olsen and Sieghart, 2009) (**Figures 1A,B**). GABA_A_Rs belong to the Cys-loop superfamily of pentameric ligand-gated ion channels (pLGICs) including strychnine-sensitive glycine receptors, nicotinic acetylcholine (nACh) receptors, and 5-hydroxytryptamine type-3 (5-HT3) receptors. Individual subunits have a common structure consisting of a large N-terminus extracellular domain (ECD) that participates in endogenous ligand binding, a transmembrane domain (TM) comprised of four α-helical regions (M1-4) and a barely extruding extracellular C-terminus. The M2 region of the subunits forms the ion channel pore. The hydrophobic M regions are connected by a small intracellular loop between M1-M2 and a much larger intracellular domain (ICD; previously termed intracellular loop) between M3 and M4 (Sigel and Steinmann, 2012) that mediates interactions with intracellular proteins critical for receptor trafficking and synaptic clustering (**Figure 1C**). Recently, GABA_A_R structures for the human β3 homopentamer bound to benzamidine (Miller and Aricescu, 2014), chimeric α5TM/β3ECD bound to the neurosteroid allopregnanolone (Miller et al., 2017), and human α1β2γ2 heteropentamer bound to GABA and the benzodiazepine site antagonist Flumazenil (Zhu et al., 2018) were resolved, advancing our growing understanding of GABA_A_R molecular architecture. Importantly, nearly all pLGIC family structural data lacks the large ICD (Nemecz et al., 2016) (exception 5-HT3 receptor; Hassaine et al., 2014), leaving functionally relevant information about this region left undiscovered.

Presynaptic terminal release of GABA onto postsynaptically clustered GABA_A_Rs initiates fast, transient receptor activation. In contrast, activation of extrasynaptic GABA_A_Rs by ambient “spill over” GABA generates a persistent tonic current (**Figure 1D**). Most GABA_A_Rs evoking fast synaptic inhibition in the mature cortex contain α1β2γ2 subunits, although α/β content can vary widely (Olsen and Sieghart, 2009), prompting a unifying role of γ2 in synaptic function. Importantly, the benzodiazepine drug class selectively binds between the interface of a γ2 subunit and either an α1/2/3/5 subunit to potentiate GABA_A_R function and elicit behavioral effects including sedative/hypnotic, anti-convulsant, myorelaxant, and/or anti-anxiety effects (Vinkers and Olivier, 2012) (**Figures 1A,B**). Here we summarize (1) known molecular interactors and mechanisms regulating γ2 trafficking (2) the importance of this subunit physiologically and human γ2 genetic variants compromising structure and function *in vitro* and *in vivo* and (3) application of modern imaging techniques to discover novel insight into synaptic GABA_A_R modulation.

## γ2 Subunit Trafficking and Interactors

### Biosynthetic Trafficking and Insertion

**FIGURE 2:**
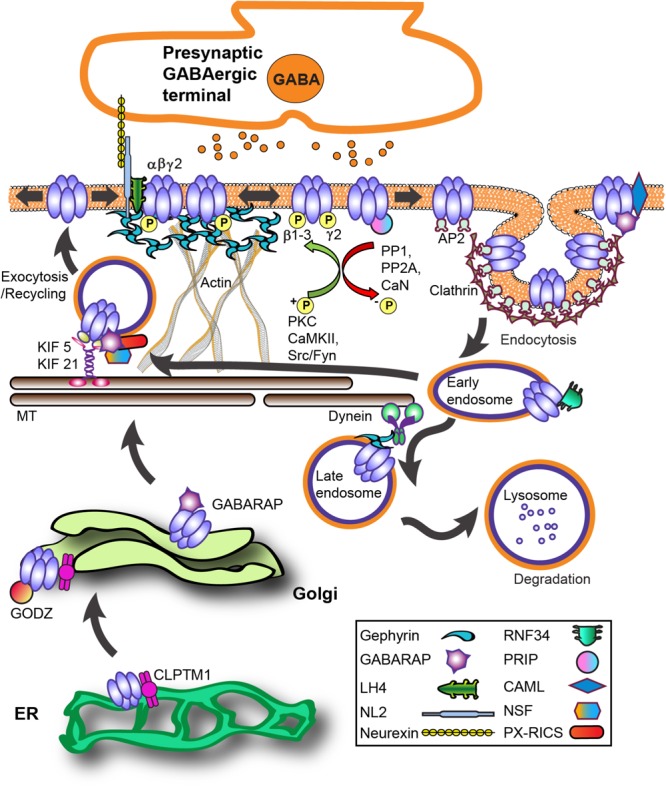
GABA_A_R trafficking and key interacting proteins at GABAergic synapses. The process of GABA_A_R synthesis, assembly and forward trafficking is highly regulated. Forward trafficking of γ2-GABA_A_Rs from the ER is negatively regulated by CLPTM1. Subunits are assembled into pentameric receptors in the endoplasmic reticulum (ER) where proper folding allows receptors to avoid proteosomal degradation and exit to the Golgi. In the Golgi, palmitoylation of γ subunits by the palmitoyltransferase GODZ is a key step in promoting forward trafficking to the synapse. GABARAP interacts with γ subunits and microtubules and overexpression augments receptor plasma membrane levels. PX-RICS forms an adaptor complex with GABARAP to facilitate γ2-GABA_A_Rs forward trafficking. PRIP1/2 and NSF interact with GABA_A_Rs both indirectly via GABARAP and directly with β subunits. The kinesin KIF5 is the main microtubule (MT)-dependent motor transporting inhibitory synapse components although recent work shows KIF21 contributes to extrasynaptic receptor delivery. LH4 forms a complex between γ2 and NL2. NL2 is central in GABA_A_R synapse development via its trans-synaptic association with axonal neurexins and also binds gephyrin. GABA_A_Rs primarily undergo clathrin-dependent endocytosis via β and γ subunit interactions with the clathrin-adaptor protein 2 (AP2) complex. Phosphorylation of AP2-interaction motifs within receptor subunits increases cell-surface receptor levels and enhances GABA_A_R neurotransmission by reducing AP2 binding to receptors. After internalization, clathrin-coated vesicles fuse with early endosomes, allowing for subsequent receptor recycling or targeting for degradation in lysosomes. CAML interaction with the γ2 subunit promotes forward trafficking and recycling. Ubiquitination of GABA_A_R contributes to lysosomal targeting, with the ubiquitin E3 ligase RNF34 directly interacting with the γ2 subunit. Protein abbreviations: CAML (calcium-modulating cyclophilin ligand), CLPTM1 (Cleft lip and palate transmembrane protein), GABARAP (GABAAR - associated protein), GODZ (Golgi-specific DHHC zinc finger enzyme), KIF 5/21 (microtubule-dependent molecular motor kinesins), LH4 (lipoma HMGIC fusion partner-like protein 4), NL2 (neuroligin 2), NSF (*N*-ethylmaleimide-sensitive factor ATPase), PRIP (phospholipase C-related catalytically inactive proteins), PX-RICS [Rho GTPase Activating Protein 32 (ARHGAP32) isoform 1], RNF34 (ring finger protein 34 E3 ligase).

During biosynthesis, GABA_A_R subunits are first assembled in the endoplasmic reticulum (ER) and then transported to the Golgi apparatus (Golgi) for further maturation (**Figure 2**). Forward trafficking of γ2-GABA_A_Rs from the ER is negatively regulated by Cleft lip and palate transmembrane protein (CLPTM1) *in vitro* and *in vivo* (**Figure 2**) (Ge et al., 2018). Overexpressing CLPTM1 reduces surface and synaptic levels of γ2, resulting in reduced amplitude and frequency of inhibitory postsynaptic current (IPSC), where the opposite effect is seen by CLPTM1 knockdown (KD). Importantly, CLPTM1 also regulates tonic inhibition and interacts with the extrasynaptic subunits α4 and δ, suggesting this protein non-selectively binds many GABA_A_R subtypes. Upon entry into the Golgi, the γ2 subunit undergoes palmitoylation via the Golgi-specific DHHC zinc finger enzyme (GODZ; also known as ZDHHC3) (Keller et al., 2004; Fang et al., 2006). This process is key for receptor clustering, innervation, and inhibitory strength *in vitro* and *in vivo* (Keller et al., 2004; Fang et al., 2006; Kilpatrick et al., 2016). GABA_A_R forward trafficking to the cell surface depends on the microtubule-dependent molecular motor kinesins (KIFs) (**Figure 2**). The KIF21B protein co-precipitates with the GABA_A_R γ2 subunit (Labonte et al., 2014). RNA KD of KIF21B reduces receptor surface levels and the intensity of extrasynaptic γ2 clusters, but does not affect synaptic GABA_A_Rs levels. Additionally, the KIF5 family plays a critical role in trans-Golgi to surface GABA_A_R trafficking (Twelvetrees et al., 2010). Conditional knockout (KO) of KIF5A in mice results in deficits of GABA_A_R plasma membrane levels, epilepsy phenotypes, and high lethality rate within 21 days postnatal (Nakajima et al., 2012).

Notably, KIF5A (not KIF5B, KIF5C) selectively interacts with the GABA_A_R-associated protein (GABARAP) *in vivo* (Nakajima et al., 2012). The well-characterized GABARAP (**Figure 2**) is part of the ubiquitin-like protein (UBL) family implicated in numerous cellular processes (van der Veen and Ploegh, 2012). GABARAP interacts with GABA_A_R γ subunits and microtubules, is heavily localized at the Golgi apparatus and cell surface (Wang et al., 1999), and overexpression augments GABA_A_R plasma membrane levels (Leil et al., 2004). However, GABARAP KO mice have unhindered distribution of γ2-GABA_A_Rs and gephyrin, suggesting functional redundancy with other trafficking proteins (O’Sullivan et al., 2005). Some evidence suggests GABARAP preferentially associates with serine phosphorylated γ2-GABA_A_Rs, while dephosphorylation by protein phosphatase 1 (PP1) decreases this interaction (Qian et al., 2011).

A number of GABARAP interacting proteins mediate GABA_A_R trafficking or localization (**Figure 2**). For instance, increased association with the PDZ domain-containing protein GRIP is seemingly involved in NMDA receptor-dependent GABA_A_R synaptic plasticity (Marsden et al., 2007). The phospholipase C-related catalytically inactive proteins 1 and 2 (PRIP1/2) and the *N*-ethylmaleimide-sensitive factor ATPase (NSF) interact with GABA_A_Rs both indirectly via GABARAP and directly with β subunits (**Figure 2**) (Kanematsu et al., 2002; Terunuma et al., 2004; Goto et al., 2005; Mizokami et al., 2007). NSF is a key component of SNARE-mediated fusion and is involved in receptor cell surface transit (Chou et al., 2010). Notably, the γ2 subunit and PRIP share an overlapping binding site on GABARAP (Kanematsu et al., 2002). PRIP1/2 KO mice demonstrate diminished benzodiazepine sensitivity and Zn^2+^ modulation concurrent with lower plasma membrane GABA_A_R expression, consistent with impaired γ2 subunit trafficking. KO of PRIP-1, the primary brain subtype, leads to mice displaying an epileptic phenotype that can be successfully suppressed by diazepam (DZP), but interictal discharges persist (Zhu et al., 2012). Interestingly, DZP potentiation of miniature inhibitory postsynaptic currents (mIPSC) remains unchanged, but baseline and DZP potentiated tonic GABA current amplitude in PRIP-1 KO neurons was reduced. PRIP-1 KO and PRIP1/2 double KO mice show anxiety-related behaviors and abnormal locomotion related to GABA_A_R dysfunction and reduced benzodiazepine sensitivity. Recently the Rho GTPase Activating Protein 32 (ARHGAP32) isoform 1 (PX-RICS) was shown to form an adaptor complex with GABARAP and the scaffold proteins 14-3-3ζ/𝜃 to facilitate γ2-GABA_A_Rs forward trafficking via dynein/dynactin and promote surface expression (Nakamura T. et al., 2016). KO of *PX-RICS* in mice generates an Autism Spectrum Disorder (ASD) phenotype with increased susceptibility to kainate-induced epileptic seizures, decreased GABA_A_R plasma membrane levels, and lowered mIPSC amplitude. Transgenic overexpression of 14-3-3ζ in mice protects against neuronal death caused by prolonged seizures (Brennan et al., 2013). In contrast, 14-3-3ζ mutations or deletions have been identified in patients with pathology associated with GABA_A_R deficits including schizophrenia, autism and generalized epilepsy (Tenney et al., 2011; Fromer et al., 2014; Toma et al., 2014).

### Synaptic Accumulation and Functional Regulation

Following insertion at the plasma membrane, γ2-GABA_A_Rs undergo Brownian diffusion until interaction with the inhibitory postsynaptic scaffolding protein gephyrin causes constraint and accumulation (**Figures 1D, 2**). Specifically, GABA_A_R α1/2/3/5 and β2/3 subunits (at lower affinity) mediate gephyrin-receptor binding (Tretter et al., 2008, 2011; Mukherjee et al., 2011; Kowalczyk et al., 2013; Brady and Jacob, 2015). While no direct interaction between γ2 and gephyrin has been identified, the synaptic levels of these proteins are intimately tied, shown by KO studies of gephyrin (Kneussel et al., 1999) and γ2 (Schweizer et al., 2003). Interestingly, chimeric studies indicate the γ2 M4 is sufficient to cause GABA_A_R accumulation opposite GABAergic terminals, while the large ICD of γ2 is necessary for gephyrin recruitment and rescue of synaptic function in γ2 KO cultured neurons (Alldred et al., 2005). It is likely that an indirect interaction occurs between γ2 and gephyrin across a bridge of other key synaptic proteins. Recently, six unrelated patients were identified with microdeletions in the gephyrin gene resulting in a range of neurodevelopmental deficits including ASD, schizophrenia or epilepsy (Lionel et al., 2013). The recently discovered GABA_A_R regulatory Lhfpl (GARLH) family proteins lipoma HMGIC fusion partner-like 3 and 4 (LH3 and LH4) forms a native complex between γ2 and the transsynaptic protein neuroligin 2 (NL2) (**Figure 2**) (Yamasaki et al., 2017). NL2 is central in GABA_A_R synapse development via its trans-synaptic association with axonal neurexins (Sudhof, 2008). Diminishing LH4 levels in culture and *in vivo* dramatically reduced γ2-GABA_A_R and gephyrin synaptic clustering and inhibitory strength (Davenport et al., 2017; Yamasaki et al., 2017). Curiously, despite the dramatic reduction in synaptic inhibition, epilepsy susceptibility or overt behavioral phenotypes in these mice have yet to be reported in the constitutive LH4 KO mouse. Importantly, gephyrin is known to directly bind the intracellular domain of NL2 (Poulopoulos et al., 2009). Thus γ2 subunit-LH4-NL2-gephyrin interactions could provide a molecular framework to support γ2’s role in GABA_A_R synaptic recruitment and maintenance.

Synaptic plasticity, or the dynamic modulation of synaptic output, is heavily influenced by receptor phosphorylation via altering channel function or receptor trafficking. Phosphoregulation of γ2 S327 is an important mediator of GABA_A_R retention at synapses. Detailed electrophysiology and *in vivo* studies have identified the PKC𝜀 isoform specifically phosphorylates the γ2 S327 residue (**Figures 1C, 2**), ultimately fine-tuning responsiveness to ethanol and benzodiazepines (Qi et al., 2007). Additionally, protocols that induce calcium-entry via glutamate application, strong NMDA receptor activation, or robust neuronal activity enhance receptor lateral mobility, decrease synaptic cluster size, and reduce mIPSC amplitude via the phosphatase calcineurin (CaN) (Bannai et al., 2009) and dephosphorylation of the γ2 subunit S327 residue (**Figures 1C, 2**) (Muir et al., 2010). More broadly, activation of all PKC isoforms by 1 h PMA (PKC activator; 30 nM) treatment decreases surface γ2-GABA_A_R levels that can be reversed by specific inhibition of PKC𝜀 catalytic activity in HEK cells and PKC𝜀 specific activation reduces GABA_A_R current amplitude (Chou et al., 2010). This effect was in part attributed to changes in GABA_A_R trafficking occurring though PKC𝜀 association and phosphorylation of NSF. The scaffolding protein 14-3-3-𝜃 acts as a bridge for the PKCγ isoform to interact with γ2 in cerebellar Purkinje neurons and N2a cells (Qian et al., 2012). 14-3-3-𝜃 KD in mice by siRNA microinjection reduces γ2-GABA_A_R overall serine phosphorylation, while KD of 14-3-3-𝜃 or PKCγ reverses the PMA (200 nM, 30 min) induced upregulation of C cell surface expression in N2a cells. These apparently conflicting reports on PKC kinase family modulation highlights the complexity of this signaling pathway in γ2-GABA_A_R regulation, with varied effects dependent on the pharmacological agents used, treatment times, model, and PKC isoforms.

An important consideration for γ2 subunit regulation is its presence in a short (γ2S) or long (γ2L) isoform; the γ2L isoform has 8 additional amino acids (LLRMFSFK) in the large ICD with the serine site (S343) capable of being phosphorylated by Protein kinase C (PKC) and Calcium/calmodulin-dependent protein kinase type II (CaMKII) (**Figure 1C**) (Whiting et al., 1990; Moss et al., 1992; McDonald and Moss, 1994). Expression levels of γ2S remain constant throughout development, while γ2L levels increase during neuronal maturation (Wang and Burt, 1991). Early *in vitro* expression studies found that the additional amino acids in the γ2L subunit may play a role in the response to diazepam and be critical for ethanol enhancement of GABA current (Wafford et al., 1991). Both mutation of S343 to a phosphomimetic aspartate or to non-phosphorylatable valine resulted in cell surface trafficking of γ2L when expressed alone, similar to γ2S (Boileau et al., 2010). This work also proposed an accessory protein role for γ2S as an external modulator of GABA_A_R function to confer zinc blockade protection for receptors. When comparing synaptic clustering of γ2L vs. γ2S subunit large ICD (partial subunit chimeras) in spinal cord neurons, postsynaptic γ2L ICD chimera accumulation is higher, and can be enhanced by PKC activation by phorbol ester phorbol-12,13-dibutyrate (PDBu) and reversed by mutating the S343 residue of γ2L (Meier and Grantyn, 2004). The physiological role of CaMKII direct phosphorylation on γ2 has not yet been described, although CaMKII is required for a type of inhibitory long term potentiation (iLTP) in Purkinje neurons known as rebound potentiation (Kano et al., 1996) and increased association between the γ2 subunit and GABARAP (Kawaguchi and Hirano, 2007). CaMKII plays other critical roles in GABAergic plasticity including promoting receptor surface levels (Wang et al., 1995; Marsden et al., 2007, 2010; Saliba et al., 2012) and recruitment of the synaptic scaffold protein gephyrin, while reducing GABA_A_R lateral diffusion (Petrini et al., 2014).

### Internalization

Non-synaptic GABA_A_Rs on the cell surface are capable of undergoing internalization (Bogdanov et al., 2006), a fundamental cellular process that regulates receptor signaling and function (**Figure 2**). GABA_A_R internalization is primarily clathrin-mediated in concert with GTPase dynamin activity and the adaptor protein AP2 complex (Kittler et al., 2000), although clathrin-independent GABA_A_R endocytosis has been described (Cinar and Barnes, 2001; Rowland et al., 2006). AP2 interacts with the ICD of GABA_A_R β subunits and the extrasynaptic δ subunit in a phospho-dependent manner (McDonald et al., 1998; Brandon et al., 2002, 2003; Herring et al., 2005; Kittler et al., 2005; Smith et al., 2008; Gonzalez et al., 2012; Smith et al., 2012). The γ2 subunit also contains two AP2 interaction domains on its ICD, a 12 basic amino acid region and a classical YGYECL motif (Smith et al., 2008) (**Figure 1C**). Phosphorylation at Y365/367 residues within the YGYECL motif by the non-receptor tyrosine-protein kinases Fyn and Src family kinases (Moss et al., 1995; Brandon et al., 2001; Jurd et al., 2010) reduces AP2 binding, as does mutation of Y365/7 to phenylalanine (Kittler et al., 2008; Tretter et al., 2009). Homozygous tyrosine to phenylalanine (Y365/7F) knock-in mice are developmentally lethal, suggesting phosphoregulation of these residues is critical for GABA_A_R function or trafficking *in vivo*. Heterozygous Y365/7F knock-in mutant mice show inhibition of AP2 binding to the γ2 subunit, surface and synaptic accumulation of receptors and ultimately spatial memory deficits (Tretter et al., 2009). Further investigation revealed that brain-derived neurotrophic factor (BDNF) enhances Y365/7 phosphorylation and stabilizes γ2-containing GABA_A_R, consistent with heterozygous Y365/7F mice showing an anti-depressant phenotype in the forced swim task and tail-suspension test and increased neurogenesis effects that are resistant to further enhancement by BDNF (Vithlani et al., 2013).

GABA_A_R endocytosis can be increased by stimuli of opposite polarities, either excitotoxic protocols such as *in vitro* seizure (Goodkin et al., 2005, 2008; Naylor et al., 2005; Lorenz-Guertin et al., 2017) and oxygen-glucose deprivation (OGD) (Arancibia-Carcamo et al., 2009), or by prolonged inhibition with agonist exposure (Chaumont et al., 2013; Gutierrez et al., 2014). Internalization is in part regulated by phosphatase activity under these conditions. For example, inhibition of CaN or the serine/threonine protein phosphatase 1 (PP1) and 2A (PP2A) reverses a status epilepticus induced decrease in surface γ2-GABA_A_Rs and mIPSC amplitude (Joshi et al., 2015). Importantly, genetic GABA_A_R mutants also affect intracellular trafficking. For instance, the γ2 R82Q (numbering without signal peptide R43Q) mutation linked to childhood absence epilepsy and febrile seizures (FS) showed increased basal receptor endocytosis rates relative to wild-type (Chaumont et al., 2013). In summary, endogenous signaling pathways, pharmacological treatments, and pathological stimuli or genetic variation can modulate GABA_A_R endocytosis networks [kinase and phosphatase regulation reviewed in Lorenz-Guertin and Jacob (2017)].

### Recycling/Lysosomal Degradation

Internalized GABA_A_Rs can either be recycled back to the cell surface or targeted for degradation at lysosomes (**Figure 2**) (Kittler et al., 2004; Arancibia-Carcamo et al., 2009). Interaction of the integral membrane protein calcium-modulating cyclophilin ligand (CAML) with the γ2 subunit cytoplasmic and fourth transmembrane domain regions promotes forward trafficking and recycling (Yuan et al., 2008). Neurons lacking CAML demonstrate diminished recycling of endocytosed GABA_A_Rs and decreased inhibitory strength. Broad PKC activity is implicated as a negative regulator of GABA_A_R recycling activity following internalization (Connolly et al., 1999). 5-HT2 serotonergic negative modulation of GABA_A_R currents is also thought to occur through a PKC-RACK1 (receptor for activated C kinase) mechanism (Feng et al., 2001).

Synaptic receptors destined for degradation undergo ubiquitination of 7 lysine residues within the ICD of the γ2 subunit (**Figure 1C**) (Arancibia-Carcamo et al., 2009). Lysine to arginine (K7R) mutation at these ubiquitination sites diminishes late endosome targeting of receptors in heterologous cells, and reverses loss of surface receptor clusters following OGD treatment (Arancibia-Carcamo et al., 2009). The ring finger protein 34 (RNF34) E3 ligase directly binds the γ2 ICD, co-immunoprecipitates with γ2 *in vivo* and can be identified at inhibitory synapses (**Figure 2**) (Jin et al., 2014). Interestingly, the short 14 amino acid motif in the γ2 ICD sufficient for RNF34 binding is identical to the GODZ binding region (**Figure 1C**), and is also highly conserved among the γ subunits. γ2-GABA_A_R degradation is accelerated upon overexpression of RNF34 resulting in smaller GABA_A_R synaptic clusters and diminished inhibitory current strength. Proteosomal and lysosomal inhibitor experiments suggest RNF34 ubiquitination of γ2 contributes to degradation by both of these pathways in HEK cells. Notably, co-expression of RNF34 with the γ2 ubiquitin resistant K7R mutant did not inhibit degradation of this subunit. On the contrary, additional lysine mutations (K8R, K9R, K10R) were able to prevent downregulation of γ2 by RNF34, suggesting these residues may be important for ubiquitination-degradation.

Only a handful of stimuli clearly induce lysosomal degradation of GABA_A_Rs, likely due to the receptor’s crucial role in maintaining neuronal inhibition and the tight regulation of receptor surface levels that must therefore occur. Our lab previously found 24 h benzodiazepine treatment in cultured hippocampal neurons enhances lysosomal-mediated degradation of α2-containing receptors (Jacob et al., 2012). More recently, we identified that a GABA_A_R antagonist bicuculline acute seizure model also induces lysosomal targeting of surface GABA_A_Rs in cultured cortical neurons (Lorenz-Guertin et al., 2017). It is likely that stimulus specific subunit ubiquitination patterns ultimately dictate receptor fate. This remains a highly understudied area of research in GABA_A_R trafficking.

### Proteomics

The network of proteins governing inhibitory synapse clustering, trafficking, and plasticity are unresolved, as evidenced by three recent *in vivo* inhibitory synapse proteomic screenings utilizing either knock-in mice expressing GFP-tagged α2 subunit (Nakamura Y. et al., 2016), adeno-associated viral (AAV) expression of fusion proteins including gephyrin (Uezu et al., 2016), or mice expressing a Thy1-His6-Flag-YFP-γ2 subunit transgene (Ge et al., 2018). Initial analysis from these experiments has revealed novel inhibitory protein constituents including the metabotropic glutamate receptor subunit mGluR5, the Dbl family GEF Ephexin, metabotropic GABA B receptor (GABA_B_R) auxiliary subunit KCTD12, and inhibitory synaptic regulator protein 1 (InSyn1) (Nakamura Y. et al., 2016; Uezu et al., 2016). Most recently, tandem affinity purification proteomics revealed the critical GABA_A_R forward trafficking component CLPTM1, and two novel interactors including integral membrane protein 2C (ITM2C) and Golgi glycoprotein 1 (GLG1) (Ge et al., 2018). Considering new candidate interactor proteins are identified with slight derivations in methodology (140 in Uezu et al., 2016; 149 in Nakamura Y. et al., 2016; 39 additional in Ge et al., 2018), future investigations will need to both confirm the validity and importance of these observed proteins in GABA_A_R function and modulation.

### Genetic Knockdown and Knockout of γ2 in Rodents

Due to the fundamental importance of γ2 GABA_A_R inhibition in the CNS, embryonic KO animals die within days of birth (Gunther et al., 1995). Developmentally delayed KO of γ2 using a CaMKIICre transgene expression system results in mice who are phenotypically normal 3 weeks post-natal, but by week 4 exhibit a rapid decline in health including epileptic episodes and eventually death (Schweizer et al., 2003). A large drop in gephyrin immunoreactivity also occurs coincident with loss of γ2 expression without changing GABAergic presynaptic innervation as measured by vesicular inhibitory amino acid transporter (VIAAT) levels.

Partial KD of brain wide γ2 levels results in impaired behavior including an enhanced anxious-depressive phenotype (Crestani et al., 1999; Chandra et al., 2005; Earnheart et al., 2007; Shen et al., 2010). In addition, heterozygous γ2^+/-^ mice show defective spine maturation and synaptogenesis (Ren et al., 2015). Ablating forebrain γ2 expression in embryonic glutamatergic neurons using homozygous EMX1Cre-induced inactivation also recapitulated the depressive-anxiety phenotype and reduced hippocampal neurogenesis similar to total heterozygous γ2 KO mice (Earnheart et al., 2007). In contrast, KD of γ2 in neurons at post-natal day 13/14 did not affect hippocampal neurogenesis, but anxiety- and depressive-like behavior still formed (Shen et al., 2012). Numerous studies have examined brain-region or cell-type specific γ2 KD or KO describing circuit specific roles that will not be discussed here (Buhr et al., 1997; Wingrove et al., 1997; Wulff et al., 2007, 2009; Lee et al., 2010; Leppa et al., 2011, 2016; Zecharia et al., 2012; Stojakovic et al., 2018).

Homozygous deletion of γ2L in mice results in near complete replacement with γ2S subunit (Homanics et al., 1999). When examining γ2 isoform specific ablation, *in vitro* findings (refer to earlier discussion in Synaptic Accumulation and Functional Regulation) would suggest GABA_A_R incorporating γ2L vs. γ2S would incur distinct changes in functional and pharmacological properties of GABA_A_R. Yet, this isoform switch did not result in changed responsiveness to ethanol in behavioral or electrophysiology experiments, although a mild increase in anxiety was observed (Homanics et al., 1999). Interestingly, the γ2L ^-/-^ mice did show a modest increase in behavioral sensitivity and GABA_A_R affinity for benzodiazepine agonists (Quinlan et al., 2000). Isoform switching of γ2 *in vivo* has been described to occur in response to certain cues such as chronic intermittent ethanol administration in rats (Petrie et al., 2001; Cagetti et al., 2003) and in schizophrenic brains of humans (Huntsman et al., 1998). The relevance of γ2 isoform switching and predominance to pathophysiology *in vivo* remains poorly understood.

## Human Genetic Variation of γ2 and Pathological Implications

### Pathology Arises From γ2 Genetic Anomalies in Humans

**FIGURE 3:**
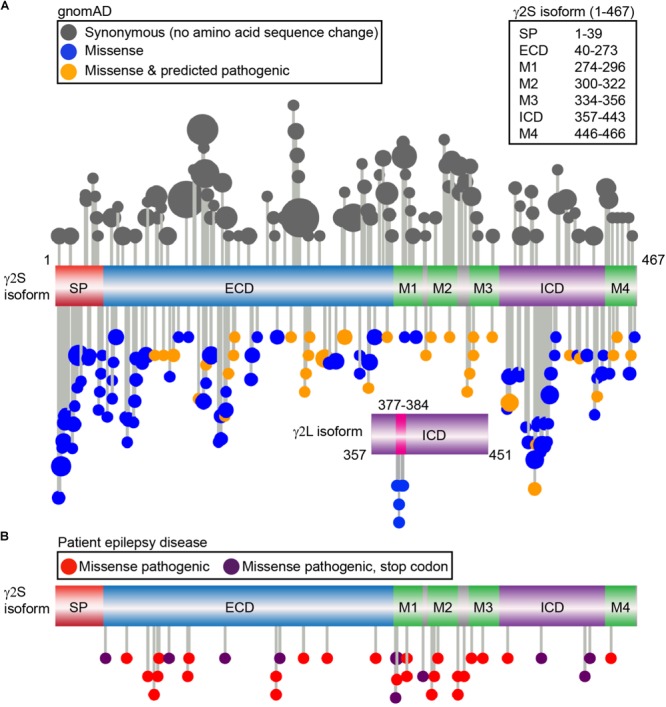
Genetic variation of the γ2 GABA_A_R subunit in gnomAD vs. genetic epilepsies. **(A)** The gnomAD dataset (individuals without any severe pediatric disease or their first-degree relatives) was used to identify a total of 104 control synonymous variants (gray) and 122 missense non-synonymous variants and plotted; each variant is represented by a lollipop marker that scales with allele frequency. Missense variants were categorized as neutral (blue) or deleterious (orange) through bioinformatics analysis using PROVEAN and SIFT predictions. Linear representation of the γ2 GABA_A_R subunit with domains: signal peptide (SP; red); extracellular N-terminal region (ECD, blue), transmembrane domain including the four transmembrane helical regions (M1-M4, green); small loops between transmembrane regions (gray); and large intracellular domain between M3-M4 (ICD, purple). The residue numbers correspond to the γ2S sequence (UniProt P18507). The independent ICD below shows the additional residues present in the γ2L isoform (UniProt P18507-2), and 5 distinct missense variants identified, predicted as neutral by PROVEAN: L377R, R379W, R379Q, F381L, S382C. **(B)** Patient epilepsy disease related missense (red) and nonsense variants leading to early stop codons (dark purple) were compiled as described in Material and Methods and plotted on the linear protein structure. All genetic variant AA residue numbering includes signal peptide.

Amongst all the subunit genes, mutations in *GABRG2* encoding the γ2 subunit are most commonly linked to epileptogenesis (Macdonald et al., 2012). Indeed, heterozygous γ2 R82Q mutant mice were one of the first *in vivo* models for childhood absence epilepsy, recapitulating a familial mutation phenotype including onset, behavior, and treatment responsiveness (Tan et al., 2007). *GABRG2* genetic anomalies including missense, nonsense, frameshift, splice-site, insertion and deletion mutations are associated with epilepsy phenotypes ranging from mild FS to moderate generalized tonic-clonic seizures or more severe disorders such as Dravet syndrome (DS) or epileptic encephalopathies (further information found in Kang and Macdonald, 2016). In order to bridge the gap between known γ2 trafficking mechanisms, identified protein interaction sites and human pathology, we examined γ2 subunit genetic variation using the Genome Aggregation Database (gnomAD) (Lek et al., 2016), a dataset of exome sequence data from 123,136 individuals and whole genome sequencing from 15,496 unrelated individuals without any severe pediatric disease and their first-degree relatives. We focused specifically on synonymous (codon substitutions result in no amino acid sequence change) and non-synonymous (alter amino acid sequence) mutations. Although synonymous codon changes were previously labeled as “silent” mutations and thought to have limited consequences, recent data indicates these may also impact function and contribute to disease through effects on *cis*-regulatory elements, mRNA structure, and protein expression. Non-synonymous mutations that result in a stop codon are referred to as nonsense mutations whereas missense mutations result in the exchange of one amino acid for another. Non-synonymous mutations may affect structural and functional properties and be associated with a disease condition; however, others may be functionally neutral and not related to a disease phenotype. Protein domains which show significant diversity in mutations identify regions of genetic flexibility, while regions with low allele frequency events (standard threshold of 0.1%) identify potentially pathogenic mutations that are not evolutionarily favored (Dudley et al., 2012). In the γ2S isoform, we identified and plotted the distribution of 104 synonymous and 122 non-synonymous missense variants (**Figure 3A**) (Jay and Brouwer, 2016). Five additional non-synonymous variants were found in the γ2L specific sequence (LLRMFSFK: L377R, R379W, R379Q, F381L, S382C), while no synonymous variants were identified (**Figure 3A**). Of note, there is a third putative γ2 isoform which appears conserved in humans and primates including the great apes and old world monkeys but absent in rodents that was not evaluated here for human genetic variation (ENST00000414552, Y211 is substituted by W, followed by 40 additional amino acids in the N-terminal extracellular domain). Overall, the latter half of the ECD, TM and linker regions showed low levels of missense variation when compared to synonymous variation (**Figure 3A**).

We next turned to the patient epilepsy disease case variants to determine if these are over-represented in similar regions. Disease case variants were gathered from National Center for Biotechnical Information (NCBI), ClinVar, and Human Gene Mutation Databases (HGMD), yielding a total of 49 pathogenic or likely pathogenic mutations including 25 missense, 11 nonsense, 9 frameshift, and 4 intron splice variants. The distribution of the 36 epilepsy-related missense and nonsense mutations was mapped across the γ2 subunit protein domains (**Figure 3B**). The 11 γ2 nonsense variants resulted in early stop codons (X) throughout the following domains: (1) ECD = Q40X, L91X, R136X, Y180X, G273X; (2) M1 = Y274X (2 unique stop codon mutant variants), W295X; (3) ICD = Q390X, R425X, W429X. The 25 γ2 subunit missense mutations showed wider distribution throughout the ECD, M1-4, M2-M3 linker and ICD regions. Comparison of the disease-associated and gnomAD missense variants identified significantly greater percentages of epilepsy related variants in the M2 and M2-M3 linker regions (**Table 1**). In contrast, signal peptide missense mutations were not found and ICD missense mutations were less prevalent in epilepsy patients (**Table 1**).

**Table 1:** Genetic variation across *GABRG2* domains.

Region	Residues	GnomAD missense	(*n* = 122)	Disease-associated missense	(*n* = 25)	*p*-value
		#	%	#	%	
Signal peptide	1–39	21	17.21	0	0.00	***^∗^0.025***
ECD	40–273	53	43.44	12	48.00	0.8255
M1	274–296	3	2.46	3	12.00	0.0616
M1-M2 loop	297–299	0	0.00	0	0.00	1
M2	300–325	2	1.64	3	12.00	***^∗^0.0348***
M2-M3 loop	326–333	2	1.64	3	12.00	***^∗^0.0348***
M3	334–356	3	2.46	2	8.00	0.2006
ICD	357–443	33	27.05	1	4.00	***^∗^0.0096***
M4	444–466	5	4.10	1	4.00	1
C-Term	467	0	0.00	0	0.00	1

*Coordinates based on *GABRG2* (GenBank NM_000816.3 transcript variant 2 γ2S, Uniprot P18507). ECD, extracellular amino-terminal domain; M1–M4, transmembrane regions 1–4; ICD, intracellular domain; C-Term, carboxy-terminus. Fisher’s exact *t-*test *p*-values are reported; ^∗^denotes statistical significance.*

In the field of medical genomics, identification of potentially pathological mutations is a significant challenge, prompting the development of multiple bioinformatics methods to assess non-synonymous variants. We used the sequence homology-based genetic analysis bioinformatics programs PROVEAN (Protein Variation Effect Analyzer) and SIFT (Sorting Intolerant from Tolerant) to assess non-synonymous variants in the gnomAD population and predict the effects on γ2 subunit biological function. Interestingly, 35 of the 122 non-synonymous gnomAD variants were also predicted to be putatively damaging/deleterious by both of the two bioinformatics tools (scoring agreement at 81.9%, **Figure 3A**, orange colored variants). Neutral scored non-synonymous variants included S386P and T388A (aka S355 and T357 phosphorylation sites, **Figure 1C**). None of the γ2L isoform missense variants were predicted by PROVEAN as damaging, although S382C (aka S343, the PKC/CaMKII phosphorylation site, see earlier Synaptic Accumulation and Functional Regulation, **Figure 1C**) was predicted as possibly damaging by SIFT. Among the gnomAD population six variants were identified that overlapped the epilepsy patient missense group (L57F, N79S, M199V, R177Q, A334T, R363Q): three were predicted as deleterious (N79S, M199V, A334T) and 3 as neutral (L57F, R177Q, R363Q). PROVEAN and SIFT bioinformatics analysis of the 25 epilepsy patient missense variants showed four as neutral (L57F, A106T, L307V and R363Q), two had conflicting predictions (L74V, R304K), and all others were scored as damaging. As the gnomAD population is relatively free from significant clinical disorders, this implies masking by epistatic genetic interactions, consistent with phenotypic variability seen in epilepsy patients and animal epilepsy models. In addition, although *in silico* prediction tools show overall robust performance, particularly when software are used in combination (Leong et al., 2015; Masica and Karchin, 2016), this suggests pathological variants can be missed. Improving clinically admissible predictions from these *in silico* tools is a current high priority focus in medical bioinformatics (Masica and Karchin, 2016; Ernst et al., 2018). To expand our insight into the cellular pathology underlying the thirty-six patient cases, we next cross-examined database information (NCBI, ClinVar, HGMD) and the current literature for disease phenotypic and cellular study based analysis.

### Patient Epilepsy Phenotypes

The most common patient phenotypes associated with nonsense and missense mutations ranged in severity and included FS, generalized tonic-clonic seizures (GTCS), GTCS with FS, genetic epilepsy with FS (GEFS), genetic epilepsy (GE), DS, and epileptic encephalopathy with severe global developmental delays (EEDD). FS are a relatively mild pathology which occur in the presence of fevers and display tonic-clonic seizure activity in individuals between 6 months and 5 years of age (Boillot et al., 2015). FS which have prolonged episode duration and occur past 6 years of age are termed FS+ and are generally associated with increased risk for developing epilepsy later in life. Moderate forms of epilepsy include GTCS and GE both with and without FS, where FS can co-occur with persistent seizure episodes past childhood and can present intense seizure activity more commonly known as a “grand mal” seizure as in the case of GTCS (Johnston et al., 2014; Wang et al., 2016; Fisher et al., 2017). The most severe phenotypes reported are DS and EEDD. In particular, DS is subset of epileptic encephalopathy and is characterized by a wide range of seizure type activity as well as psychomotor development delays, ataxia and hyperkinesis emerging between the ages of 1–4 (Ishii et al., 2014; Fisher et al., 2017). In contrast, EEDD have broader phenotypic manifestations and deficits as a result of global neurodevelopmental impairments with treatment-resistant seizures (Shen et al., 2017). Less common reported patient phenotypes included myoclonic epilepsy, absence seizures, complex partial seizures, tonic infantile spasms, tonic seizures, Rolandic epilepsy, and ASD with learning difficulties. *In vitro* studies have been invaluable in gaining in depth understanding of etiology, cellular pathology, and functional effects of these epilepsy patient variants.

### γ2 Subunit Disease Case Analysis

*In vitro* studies on 17 of the γ2 pathogenic variants have revealed reduced surface expression in 15 cases, in part resulting from ER retention and trafficking defects (**Table 2**). The severe disease DS epilepsy phenotype is associated with three nonsense mutations (Q40X, R136X, Q390X) and one missense (P302L) mutation (**Table 2**). The early occurrence of Q40X and R136X within the ECD resulted in premature termination codons (PTCs) and mRNA degradation via nonsense mediated mRNA decay (NMD) with decreased γ2 protein levels. The introduction of upstream PTCs limited the availability of trafficable γ2, diminished overall receptor surface expression and synaptic localization and resulted in significant GABAergic deficits (Ishii et al., 2014). Conversely, the Q390X (previously known as Q351X) mutation occurs in the ICD and escapes NMD but is instead subject to ubiquitin-proteasome degradation (Kang et al., 2013). *In vitro* experiments found Q390X to have comparable mRNA levels to other late sequence nonsense mutations but dissimilar protein expression due to different degradation rates. Q390X displayed a substantially longer half-life as compared to wild-type γ2 and other nonsense mutant subunits in addition to an increased ability to oligomerize with and sequester wild-type α and β subunits. This alternative disruption in receptor trafficking provides evidence that expressed non-functional truncated subunits may be modifiers of epilepsy phenotype severity. Interestingly, P302L was the only missense mutation reported in a patient with DS (Hernandez et al., 2017). Of note, this mutation resides in M2 and contributes to the formation of the ion channel pore which likely explains its severe phenotype. This is supported by P302L mutant electrophysiological studies and structural modeling which suggests a shift in pore activity resulting in slow activation, low conductance states, and fast desensitization of GABA_A_R (Hernandez et al., 2017). In contrast, all six cases of EEDD were found in patients with missense mutations (A106T, I107T, P282S, R323W, R323Q, F343L) dispersed throughout structural domains (ECD, M1 and M2) and exhibited additional epileptic phenotypes such as GTCS, GEFS, and tonic seizures (Shen et al., 2017). In fact, the I107T mutation is located in the ECD which typically tolerates missense mutations as evidenced by relatively mild phenotypes; however, this mutation was found to exhibit the most severe cellular pathologies as compared to other disease variants emphasizing the need to further investigate these mutations and their ramifications on cellular processes.

**Table 2:** *GABRG2* missense and nonsense patient mutations with associated cellular pathologies and reported clinical phenotypes.

	Cellular Pathalogy					Phenotype						Publication(s)
Region	Variant	Trafficking	ER retention	Surface expression	Cell current	Febrile seizures	Generalized tonic-clonic seizures	Genetic epilepsy	Dravet syndrome	Epileptic encephalopathy	Other	
ECD	Q40X	↓↓	↑	↓↓	↓↓				✓			Hirose et al., 2005; Huang et al., 2012; Macdonald et al., 2012; Ishii et al., 2014
ECD	L57F	–	✓	–	↓↓			✓				Hernandez et al., 2016
ECD	N79S	↓	–	↓	↓		✓					Shi et al., 2010; Migita et al., 2013; Huang et al., 2014
ECD	R82Q	↓	↑	↓	↓	✓					✓	Wallace et al., 2001; Bianchi et al., 2002; Bowser et al., 2002; Macdonald et al., 2003; Kang and Macdonald, 2004; Sancar and Czajkowski, 2004; Kang et al., 2006; Eugene et al., 2007; Frugier et al., 2007; Macdonald et al., 2012; Chaumont et al., 2013; Bennett et al., 2017
ECD	P83S	↓↓	↑	↓↓	↓↓	✓		✓				Lachance-Touchette et al., 2011; Huang et al., 2014; Bennett et al., 2017
ECD	A106T	↓↓	↑	↓↓	↓↓		✓			✓	✓	Shen et al., 2017
ECD	I107T	↓↓	↑	↓↓	↓↓					✓	✓	Shen et al., 2017
ECD	R136X	↓↓	↑	↓↓	↓↓	✓		✓	✓		✓	Kang et al., 2013; Johnston et al., 2014
ECD	G257R	↓↓	↑	↓↓	–						✓	Reinthaler et al., 2015
M1	P282S	↓↓	↑	↓↓	↓↓					✓		Shen et al., 2017
M2	P302L	–	↔	↓	↓				✓			Hernandez et al., 2017
M2	R323W	↓↓	↑	↓↓	↓↓		✓			✓	✓	Shen et al., 2017
M2	R323Q	↓↓	↑	↓↓	↓↓	✓	✓	✓		✓	✓	Carvill et al., 2013; Reinthaler et al., 2015; Shen et al., 2017
M2-M3 loop	K328M	↓↓	↑	↓↓	↓↓	✓		✓				Baulac et al., 2001; Bianchi et al., 2002; Macdonald et al., 2003, 2012; Ramakrishnan and Hess, 2004; Hirose et al., 2005; Kang et al., 2006; Eugene et al., 2007; Frugier et al., 2007; Bouthour et al., 2012; Bennett et al., 2017
M3	F343L	↓↓	↑	↓↓	↓↓					✓	✓	Shen et al., 2017
ICD	Q390X	↓↓	↑	↓↓	↓↓	✓		✓	✓			Singh et al., 1999; Harkin et al., 2002; Kang et al., 2006, 2009; Macdonald et al., 2012; Kang et al., 2013
ICD	W429X	↓↓	↑	↓↓	↓↓	✓		✓				Sun et al., 2008; Macdonald et al., 2012; Kang et al., 2013; Wang et al., 2016

*Variants are organized by nucleotide sequence position, and variants ending in ‘X’ are nonsense mutations with all others being missense mutations. Other epileptic or developmental phenotypes include absence seizures, complex partial seizures, severe global developmental delay, tonic infantile spasms, autism disorder with learning difficulties, Rolandic epilepsy, or epilepsy with myoclonic-astatic seizures; ECD, extracellular amino-terminal domain; M1–M3, transmembrane regions 1–3; ICD, intracellular domain; ↓↓, reduced; ↑, increased; ✓, observed; ✓, unknown; -, not affected/changed; ↓, slightly reduced; ↔, possibly.*

The moderate epileptic phenotype GEFS without co-occurring conditions was observed in three cases with two missense (P83S and K328M) and one nonsense (W429X) variants reported with structural locations in the ECD, M2-M3 linker, and ICD, respectively (**Table 2**). P83S was found to reduce GABA-evoked whole cell currents mainly through a plasma membrane and trafficking-dependent manner (Lachance-Touchette et al., 2011; Huang et al., 2014; Bennett et al., 2017). In contrast, K328M (previously known as K289M) is found in the short extracellular loop between the M2-M3 regions and was found to increase receptor deactivation, implicating this region in receptor kinetic properties (Macdonald et al., 2012). Conversely, W429X displayed less drastic protein degradation and subunit oligomerization pathologies compared to the previously discussed DS variant Q390X (Wang et al., 2016). The later downstream incidence of W429X combined with slightly higher surface expression compared to Q390X may explain the milder epilepsy phenotype (Sun et al., 2008; Macdonald et al., 2012; Kang et al., 2013; Wang et al., 2016).

Throughout the reviewed mutations, only two variants (L57F and N79S) deviated from a pathology associated with reduced γ2 containing GABA_A_R plasma membrane levels and were located in the ECD. L57F was present in an individual with GE and found to have normal surface and trafficking characteristics compared to wild-type γ2 receptors; however, altered current density properties and function were observed possibly due to minor structural perturbations in the α1-helix of the ECD (Hernandez et al., 2016). Comparatively, the N79S mutation was the sole occurrence of GTCS without co-occurring phenotypes and presented slight but significant impairments in plasma membrane levels and peak current amplitude (Huang et al., 2014) suggesting it is more of a susceptibility variant as opposed to an epilepsy mutation (Shi et al., 2010; Migita et al., 2013; Huang et al., 2014). Moreover, the resilience of the ECD is further supported by R82Q (previously known as R43Q), a well characterized missense mutation associated with mild phenotypic manifestations like FS and absence seizures with trafficking deficient pathologies (Macdonald et al., 2012). Overall, the 13 frameshift and intron splice variant mutations analyzed were associated with mild phenotypes, though further studies are needed to elucidate their pathological mechanisms (**Table 3**). However, frameshift mutations within the ICD (E402Dfs^∗^3 generating a stop codon at Y404X critical Src/Fyn phospho site discussed earlier; and S443delC resulting in an altered and elongated carboxy terminus with +50 novel AA) were associated with more moderate-severe phenotypes like GTCS and GEFS+ underscoring the importance for intracellular regulation via the ICD (Macdonald et al., 2012).

**Table 3:** Patient frameshift mutations and intron splice variants associated or likely associated with various epilepsy phenotypes.

Region	Canonical sequence codon	Mutant sequence	Variant name	Mutation type	Phenotype(s)	Function effect(s)
ECD	ACT-CCA-AAA 58 59 60	ACA-CAA-AAG	P59Qfs^∗^12	Frame shift	Febrile Seizures, Tonic-Clonic Seizures	Predicted to undergo NMD (Boillot et al., 2015).
ECD	TTT-GCG-CAA 117 118 119	TTT-TGC-GCA	A118Cfs^∗^6	Frame shift	Febrile Seizures	Predicted to undergo NMD (Della Mina et al., 2015).
ECD	AAA-GCT-GAT 57 58 59	AAG-CTG-ATG	A158Lfs^∗^13	Frame shift	Unknown	Predicted to cause loss of normal protein function either through protein truncation or NMD. #
ECD	CGA-GTG-CTC 177 178 179	CAG-TGC-TCT	R177Qfs^∗^6	Frame shift	Childhood Absence Epilepsy, Febrile Seizures	Predicted to cause loss of normal protein function either through protein truncation or NMD. #
Intron 4	CTT-AGG-TTG Int4 Int4 184	CTG-AGG-TTG	549-3T > G	Intron Splice Variant	Unknown	Abnormal gene splicing; *in silico* assessment predicts altered protein function (Reinthaler et al., 2015).
Intron 6	TCC-GTG-AAG 256 Int6 Int6	TCC-GGG-AAG	IVS6 + 2T– > G	Intron Splice Variant	Childhood Absence Epilepsy, Febrile Seizures	Truncation; ER retention; undergo NMD; decreased surface γ2 subunit levels and GABA-evoked whole cell currents; and increased ER stress marker BIP (Kananura et al., 2002; Tian and Macdonald, 2012).
ECD	GGA-GAT-TAT 257 258 259	AGA-GAT-TAT	770-1G > A	Intron Splice Variant	Suspected to cause epilepsy	Predicted to cause abnormal gene splicing and undergo NMD or the production of an abnormal protein. #
M3	GTT-TGT-TTC 341 342 343	GTT-TTT-TCA	C342Ffs^∗^50	Frame shift	Childhood Absence Epilepsy, Febrile Seizures	Not anticipated to result in NMD but expected to result in a truncated protein. #
FproveIntron 8	CAG-GCC-CCT Int8 377 378	CGG-GCC-CCT	1129-2A > G	Intron Splice Variant	Childhood Absence Epilepsy, Febrile Seizures	Not anticipated to undergo NMD, but likely alters RNA splicing and disrupts protein function. #
ICD	ATT-CAA-GAG 397 398 399	ATT-CGA-GAG	Q398Rfs^∗^4	Frame shift	Unknown	Predicted to cause protein truncation. #
ICD	GAA-GAG-TAC 402 403 404	GAT-TCA-TGA	E402Dfs^∗^3	Frame shift	Febrile Seizures, Temporal Lobe Encephalopathy, Generalized Tonic-Clonic Seizures, Focal seizures	Predicted to cause protein truncation (Boillot et al., 2015). #
ICD	TCC-TAT-GCT 443 444 445	TCT-ATG-TCT	S443delC	Frame shift	Genetic Epilepsy with Febrile Seizures Plus	Produced elongated peptide with 50 novel amino acids compared to γ2S; trafficking impairments, ER retention, decreased surface expression and whole cell currents (Tian et al., 2013).
M4	GTC-TCC-TAC 462 463 464	TCT-CCT-ACC	V462Sfs^∗^33	Frame shift	Febrile Seizures	Predicted to escape NMD and produce elongated peptide with 32 novel amino acids as compared to γ2S (Boillot et al., 2015). #

*Patient variants are ordered by nucleotide sequence position of *GABRG2*. Nucleotides deleted (red) and inserted (green) for each variant are noted. ECD, extracellular amino-terminal domain; M3-M4, transmembrane regions 3-4; ICD, intracellular domain; NMD, nonsense-mediated mRNA decay; introduction of downstream premature stop codon following specified number of codons (^∗^); predicted function from GeneDX (#).*

In summary, both deficits in GABA_A_R surface trafficking and the functional role of specific γ2 subunit regions are critical factors modulating phenotypic outcome, with some missense mutations resulting in phenotypes as severe as nonsense mutations. Furthermore, expressed non-functional truncated subunits may be correlated with more severe manifestations and be modifiers of disease phenotypes. Disease case variants in the pore lining M2 region showed particularly severe phenotypes, consistent with the reduced genetic variation in this region in gnomAD non-synonymous variants. Clearly, *in vitro* studies of recombinant receptor trafficking, electrophysiology and assembly have provided important insight into the underlying cellular pathology and functional effects of these epilepsy patient variants. Greater understanding of the consequences of γ2 genetic variation, both for revealing disease mechanisms and for GABA_A_R synaptic plasticity will be gained through application of innovative imaging methods in the neuronal context.

## Looking Forward: Imaging Advances

Advancing imaging techniques are providing critical insight into GABA_A_R trafficking extending beyond basic endo/exocytic trafficking of receptors. Live-cell imaging using pH-sensitive GFP (pHluorin) tagged GABA_A_Rs subunits and fluorescence recovery after photobleaching (FRAP) experiments first identified GABA_A_R synaptic retention, limiting diffusion at synaptic release sites, and the crucial role of gephyrin in this process (Jacob et al., 2005). Receptor subunits with pHluorin tags have further described GABA_A_R surface levels and lysosomal degradation (Jacob et al., 2012; Lorenz-Guertin et al., 2017) and novel exocytic machinery and insertion sites of receptors (Gu et al., 2016). The pHluorin-FRAP technique is often performed in addition to the newer workhorse of diffusion studies, quantum dot (QD) single-particle tracking. QD studies have revealed precise quantitative properties of synaptic and extrasynaptic GABA_A_R diffusion during baseline conditions (Renner et al., 2012), excitatory stimulation (including iLTP) (Bannai et al., 2009, 2015; Muir et al., 2010; Niwa et al., 2012; Muir and Kittler, 2014; Petrini et al., 2014), GABA_A_R agonist and/or drug treatment (Gouzer et al., 2014; Levi et al., 2015; de Luca et al., 2017), GABA_B_ receptor activation (Gerrow and Triller, 2014), purinergic (P2x2 receptor) activation (Shrivastava et al., 2011), and changes in gephyrin or radixin phosphorylation (Hausrat et al., 2015; Battaglia et al., 2018). Receptor functional regulation by changes in surface diffusion, perhaps completely independent of changes in surface levels, represents a paradigm shift in our basic understanding of synaptic plasticity. Indeed current studies of human genetic variants in recombinant systems are unlikely to detect these fundamentally important properties due to lack of a neuronal context, the appropriate GABA_A_R subunit complement, interacting proteins, and general overexpression problems. For example, QD neuronal studies of the γ2 K328M disease variant revealed an additional phenotype of enhanced temperature sensitive receptor diffusion, likely contributing to the FS pathology in patients (Bouthour et al., 2012).

To address multiple trafficking questions within a single assay, our group recently engineered a GABA_A_R γ2 subunit dual fluorescent sensor encoding a pHluorin tag and a fluorogen-activating peptide (FAP) (γ2^pH^FAP) (Lorenz-Guertin et al., 2017). FAPs are antibody single chain variable fragments characterized to selectively bind inorganic dyes with high specificity and affinity (Szent-Gyorgyi et al., 2008). The dyes are non-fluorescent until bound by a FAP and individual dyes have unique characteristics including cell permeability, pH-sensitivity, fluorescent properties, and *in vivo* administration capability (Fisher et al., 2010; Grover et al., 2012; Saunders et al., 2012; Zhang et al., 2015; He et al., 2016). We have used the FAP-dye system in neurons to selectively examine cell surface GABA_A_Rs undergoing internalization, early endosomal accumulation and targeting to late endosomes/lysosomes via confocal live-imaging (Lorenz-Guertin et al., 2017). Pulse-labeling γ2^pH^FAP with cell impermeable dye allows for detection of surface receptor turnover rates independent of a change in total GABA_A_R surface levels, as we demonstrated using a mild seizure protocol. As more GABA_A_Rs subunits are engineered to express the FAP tag, and additional unique dyes are synthesized to address specific experimental questions, the utility of this imaging approach continues to grow.

Other innovative imaging approaches advancing our ability to detect changes in GABA_A_R synaptic plasticity include optogenetic toolkits for controlling GABA_A_R activity (Lin et al., 2014, 2015), spatially regulated GABA activation using two-photon photolysis (Oh et al., 2016), proximity ligation assays to measure endogenous protein interaction (Smith et al., 2014; Tseng et al., 2015; Ghosh et al., 2016), and super-resolution imaging and other fluorescent tools to examine inhibitory gephyrin scaffolding (Gross et al., 2013, 2016; Sigal et al., 2015; Maric et al., 2017; Pennacchietti et al., 2017). Fluorescence resonance energy transfer (FRET) techniques have been limitedly applied to studying GABA_A_R trafficking or receptor subunit composition (Ding et al., 2010; Shrivastava et al., 2011), collectively suggesting imaging techniques will be a rich resource of novel GABA_A_R knowledge.

## Conclusion

In summary, we live in an unprecedented time for understanding human disease pathology and neurodevelopment through integration of “big data” on human genetic variation and protein interaction networks/interactomes, in combination with high resolution live-imaging approaches. Future efforts to resolve GABA_A_R pathologies will benefit from connecting genetic variants to their cellular mechanisms of pathology within the complexity of neuronal signaling. Importantly, increased understanding of surface and intracellular pool regulated trafficking of GABA_A_R will provide mechanisms to treat overall reduced receptor levels in various disease states. Future treatment of genetic epilepsy syndromes are likely to involve CRISPR-Cas9 gene editing (Ma et al., 2017), RNA focused REPAIR editing approaches, or application of improved drugs that act as chaperones to promote receptor trafficking. The new imaging based methods described here are particularly likely to show high utility in both identifying cellular pathology of human GABA_A_R genetic variants and for drug screening efforts in a neuronal context.

## Materials and Methods

### Data Mining of *GABRG2* Genetic Variation

The prevalence of γ2 subunit non-synonymous and synonymous variations in gnomAD^1^, currently a dataset of exome sequence data from 123,136 individuals and whole genome sequencing from 15,496 unrelated individuals, was assessed and restricted to those meeting the “PASS” quality threshold (Lek et al., 2016). Individuals known to be affected by severe pediatric disease are not contained in this data set, or their first-degree relatives. Next “pathogenic” and “likely pathogenic” patient case variants not present in the gnomAD dataset were investigated in National Center for Biotechnical Information variation viewer (NCBIvv)^1^, ClinVar, and Human Gene Mutation Databases (HGMD) utilizing the following search parameters: GRCh37.p13 annotation release 105 assembly and NM_000816.3 (transcript variant 2, γ2S). The search in NCBIvv identified 17 variants (accessed January 2018). The ClinVar search (accessed February 2018) confirmed 16/17 candidate variants with the outlier (R323W) having been newly identified in the literature (Shen et al., 2017)^2^. In addition to those confirmed, the ClinVar investigation produced 10 additional mutations. Some variants identified in ClinVar had associated predicted functions (submitted by GeneDX genetics company)^3^. Finally, HGMD (hgmd2018.1; accessed March 2018) interrogation uncovered 22 disease-causing mutations that were absent from NCBIvv and ClinVar inquiries^4^. Using these candidate case variants and their associated database information, the current literature was evaluated for disease phenotypic and cellular study based implications yielding a total of 49 pathogenic or likely pathogenic mutations including 25 missense, 11 nonsense, 9 frameshift, and 4 intron splice variants. We used lollipops-v.1.3.1 software (Jay and Brouwer, 2016) to plot the distribution of synonymous, non-synonymous and disease case mutations in *GABRG2* along a linear γ2S assembly (P18507, ENST00000361925) and a linear segment representation of the additional eight encoded amino acids within the ICD in the γ2L isoform (P18507-2, ENST00000356592). The missense and nonsense disease case variants studied at the cellular trafficking level were included in **Table 2**. The frameshift and intron splice variants were annotated in **Table 3**.

### Bioinformatics Tools

PROVEAN (Protein Variation Effect Analyzer^5^ (Choi et al., 2012) and SIFT (Sorting Intolerant from Tolerant) algorithms (Hu and Ng, 2013) are bioinformatics tools which predict whether an amino acid substitution or indel (insertion or deletion) has an impact on a protein’s biological function using homology based genetic analysis. Currently PROVEAN provides scoring via both PROVEAN and SIFT algorithms. PROVEAN utilizes pairwise sequence alignment scores to generate pre-computed predictions at every amino acid position in all human and mouse protein sequences. Mutations are predicted to be deleterious or tolerant based on the prediction cutoff value of -2.5: scores smaller than -2.5 are considered deleterious. Similarly, SIFT predicts whether the amino acid substitution alter the protein function based on sequence homology and the physical properties of amino acids. The intolerant range of SIFT is ≤0.05 for predicted damaging/deleterious mutations and a score of >0.05 predicts the tolerant range.

## Web Resources

• gnomAD, http://gnomad.broadinstitute.org/
• ClinVar, https://www.ncbi.nlm.nih.gov/clinvar/
• Human Gene Mutation Database, http://www.hgmd.org/
• Lollipops v.1.3.1, https://github.com/pbnjay/lollipops/releases
• UniProt, http://www.uniprot.org/

## Author Contributions

JL-G and TJ wrote and edited the sections “γ2 Subunit Trafficking and Interactors” and “Looking Forward: Imaging Advances.” MB and TJ analyzed, wrote, and prepared the section “Human Genetic Variation of γ2 and Pathological Implications” and associated tables. TJ prepared all the figures. MB prepared all the tables.

## Conflict of Interest Statement

The authors declare that the research was conducted in the absence of any commercial or financial relationships that could be construed as a potential conflict of interest. The reviewer MG and handling Editor declared their shared affiliation at the time of review.

## Footnotes

1 http://gnomad.broadinstitute.org/transcript/ENST00000361925

2 https://www.ncbi.nlm.nih.gov/clinvar

3 https://www.genedx.com/

4 http://www.hgmd.cf.ac.uk/ac/gene.php?gene=GABRG2

5 http://provean.jcvi.org
